# Cyanidin-3-glucoside inhibits ferroptosis in renal tubular cells after ischemia/reperfusion injury via the AMPK pathway

**DOI:** 10.1186/s10020-023-00642-5

**Published:** 2023-04-03

**Authors:** Yi-Wei Du, Xiao-Kang Li, Ting-Ting Wang, Lu Zhou, Hui-Rong Li, Lan Feng, Heng Ma, Hong-Bao Liu

**Affiliations:** 1 Department of Nephrology, Tangdu Hospital, Air Force Military Medical University (Fourth Military Medical University), Xi’an, 710038 China; 2 Department of Cardiology, Tangdu Hospital, Air Force Military Medical University (Fourth Military Medical University), Xi’an, 710038 China; 3 Department of Physiology and Pathophysiology, School of Basic Medical Sciences, Air Force Military Medical University (Fourth Military Medical University), Xi’an, 710038 China

**Keywords:** Acute kidney injury (AKI), AMP-activated protein kinase (AMPK), Cyanidin-3-glucoside (C3G), Ferroptosis, Ischemia, Lipid peroxidation

## Abstract

**Background:**

Ferroptosis, which is characterized by lipid peroxidation and iron accumulation, is closely associated with the pathogenesis of acute renal injury (AKI). Cyanidin-3-glucoside (C3G), a typical flavonoid that has anti-inflammatory and antioxidant effects on ischemia‒reperfusion (I/R) injury, can induce AMP-activated protein kinase (AMPK) activation. This study aimed to show that C3G exerts nephroprotective effects against I/R-AKI related ferroptosis by regulating the AMPK pathway.

**Methods:**

Hypoxia/reoxygenation (H/R)-induced HK-2 cells and I/R-AKI mice were treated with C3G with or without inhibiting AMPK. The level of intracellular free iron, the expression of the ferroptosis-related proteins acyl-CoA synthetase long chain family member 4 (ACSL4) and glutathione peroxidase 4 (GPX4), and the levels of the lipid peroxidation markers 4-hydroxynonenal (4-HNE), lipid reactive oxygen species (ROS) and malondialdehyde (MDA) were examined.

**Results:**

We observed the inhibitory effect of C3G on ferroptosis in vitro and in vivo, which was characterized by the reversion of excessive intracellular free iron accumulation, a decrease in 4-HNE, lipid ROS, MDA levels and ACSL4 expression, and an increase in GPX4 expression and glutathione (GSH) levels. Notably, the inhibition of AMPK by CC significantly abrogated the nephroprotective effect of C3G on I/R-AKI models in vivo and in vitro.

**Conclusion:**

Our results provide new insight into the nephroprotective effect of C3G on acute I/R-AKI by inhibiting ferroptosis by activating the AMPK pathway.

## Background

Acute kidney injury (AKI), which has high mortality and morbidity, remains a threat to patients due to inefficient treatments (Ronco et al. 2019). Ischemia‒reperfusion (I/R) is one of the major causes of AKI, and renal tubular epithelial cells (RTECs) are susceptible to hypoxia and other injuries. Diverse forms of cell death, such as necrosis and apoptosis are known to contribute to RTEC damage and kidney dysfunction (Pefanis et al. 2019; Zhao et al. 2020). Ferroptosis, a recent term coined in 2012 for another type of regulated cell death, is characterized by the inactivation of the antioxidant defensive system, imbalanced iron ions, and the subsequent accumulation of lipid peroxidation (Jiang et al. 2021; Ni et al. 2022). Ample evidence has shown that ferroptosis plays a pivotal role in I/R-AKI, and it predominantly contributes to RTEC death predominantly compared to regulated programmed cell death (Pefanis et al. 2019; Zhao et al. 2020; Ni et al. 2022; Linkermann et al. 2014; Martin-Sanchez et al. 2017). Overall, targeting ferroptosis can be a therapeutic strategy to ameliorate I/R-AKI, however, there is still a lack of effective drugs for ferroptosis in the clinic.

Cyanidin-3-glucoside (C3G), which is a type of anthocyanin, is a member of a typical subgroup of soluble flavonoids. It has been reported that C3G can attenuate acute organ injury by increasing oxidation resistance and glutathione (GSH) levels, in lipopolysaccharide-induced acute lung injury (Tsuda et al. 1999; Ma et al. 2015; Yan et al. 2015), heart ischemic injury (Fu et al. 2014; Li et al. 2021; Trinei et al. 2022), and diabetic nephropathy (Skemiene et al. 2015). Moreover, C3G can modulate oxidation mediated by iron and chelated iron (Toyokuni et al. 2002; Qin et al. 2018a; Xie et al. 2018; Zhang et al. 2020). Thus, we hypothesized that C3G could be a promising candidate for inhibiting ferroptosis with potential implications in the treatment of renal I/R injury.

RTECs require large quantities of ATP to achieve their transport function, especially when exposed to hypoxia and reoxygenation (H/R) (Pefanis et al. 2019). AMP-activated protein kinase (AMPK), which modulates cellular metabolism and restores homeostasis as an energy switch, senses the change of energy status in cells (Bhargava and Schnellmann 2017; Herzig and Shaw 2018; Lin and Hardie 2018), and participates in metabolic conditions in the kidney. Inactivation or deletion of AMPK can exacerbate acute I/R injury in the kidney, resulting in morphological changes and biochemical indicators (Lee et al. 2020). Recently, the close correlations between AMPK and ferroptosis were investigated in many cell lines and diverse disease models, revealing distinct outcomes in which AMPK acts as an inducer or an inhibitor of ferroptosis (Lee et al. 2020; Song et al. 2018; Han et al. 2020; Wang et al. 2020). However, studies about C3G affects ferroptosis by regulating AMPK has not been investigated.

C3G is a kind of food additive, and the safety of in humans was evaluated in a clinical trial (Mertens-Talcott et al. 2008; Qin et al. 2009). Intriguingly, dietary intake of anthocyanins can reduce the risk of chronic disease(Qin et al. 2018a; McCullough et al. 2012). Thus, in this study, we examined the hitherto unrecognized coupling among C3G, AMPK, and ferroptosis, providing reliable evidence for the research and development of renal protective drugs for the clinical treatment of AKI.

## Methods

### Chemicals and reagents

Cyanidin-3-glucoside (C3G, #C832095, with the purity ≥ 98%) was purchased from Macklin (Shanghai, China). Erastin (Era) (HY-15763) and Compound C (CC) (HY-13418A) were purchased from Med Chem Express (Shanghai, China). Liproxstatin-1 (Lip-1), ferroptosis inhibitor, was bought from Selleck (S7699, Selleck, TX, USA). In vitro experiments, the following reagents were used at the appropriate concentrations: Era (1 μM) and CC (10 μM) for HK-2 cells. C3G (50 μM) or Lip-1(0.5 μM) were added, respectively, and incubated with Era. According to the manufacturer and refer to published literatures (Shan et al. 2021; Krama et al. 2022; Li et al. 2020; Qin et al. 2018b), we finally determined a relatively low dosage and timing of C3G and final concentration and timing of CC. For in vivo experiments, the dosage of the drugs was C3G (10 mg/kg) and CC (10 mg/kg). C3G was intraperitoneal injection daily for a week with or without intravenous injection of CC once 30 min before I/R. C3G was dissolved in dimethyl sulfoxide (DMSO) and then diluted with saline to make sure DMSO concentration was less than 0.1% (v/v), just like previously described (Shan et al. 2021).

### Animals and experimental protocols

All animal experiments were carried out in strict accordance with the Guidelines of Health and guidelines for use, permitted by the Scientific Investigation Committee of the Air Force Medical University. Male C57BL/6 mice (6–8 weeks, weight 20–25 g) were purchased from Experimental Animal Center of the Air Force Medical University (Xi'an, China), after one week of adaption, they were randomly divided into four groups (n = 6 for each group): sham group (equivalent saline containing 0.1% DMSO), I/R group (both renal pedicles were clamped for 33 min using nontraumatic microaneurysm clips), sham + C3G group (intraperitoneal injection of 10 mg/kg C3G), and I/R + C3G group (intraperitoneal injection of 10 mg/kg C3G).

### Renal function assays and histology

Tissues and samples were collected immediately at the time of euthanasia after reperfusion for 24 h, in which the whole blood obtained from the retroocular vein plexus was centrifuged at 4 °C, 3000 rpm, for 15 min to acquire the serum sample and kidneys were fixed in 4% phosphate-buffered formaldehyde and frozen respectively for different assays. Subsequently, we detected the level of blood urea nitrogen (BUN) and serum creatinine (Scr) according to the manufacturer’s instructions using the urea determination kit (C013-2–1, Nanjing Jiancheng, China) and creatinine determination kit (C011-2–1, Nanjing Jiancheng, China). Half of one kidney was embedded in paraffin, cut into 4 μm thick sections, and stained with Hematoxylin–eosin (HE) and Periodic acid-Schiff (PAS).

### Detection of ROS

The level of ROS was detected by dihydroethidium (DHE) fluorescent probe and tissue sections were incubated in 4 μM DHE for 1 h at 37 °C in a humidified chamber protected from light. Owing to the presence of superoxide anion, DHE is oxidized to ethidium, indicating a bright red fluorescence.

### Immunofluorescence and immunohistochemistry

The paraffin kidney sections were applied in immunofluorescence and immunohistochemistry staining to determining the expression levels of various proteins. Primary antibodies for acyl-CoA synthetase long chain family member 4 (ACSL4) (1:200, DF12141, Affinity), glutathione peroxidase 4 (GPX4) (1:200, DF6701, Affinity), 4-hydroxynonenal (4-HNE) (1;100, MAB3249, R&D Systems). Next, the slides were exposed to DAB-labeled secondary antibodies, washed with deionized distilled water, and ultimately observed by light microscopy.

### Cell culture, treatment and transfection

The human renal proximal tubular cell line HK-2 was purchased from the China Center for Type Culture Collection (GDC0152, Wuhan, China), and was cultured in Dulbecco’s modified Eagle’s medium/nutrient mixture F-12 (DMEM/F-12) supplemented with 10% fetal bovine serum (FBS), 100 μg/ml penicillin, and 100 μg/ml streptomycin in a humidified atmosphere of 5% CO_2_ and 95% air at 37 °C. NRK-52E cells were obtained from the China Cell Bank (GNR 8, Shanghai, China), and grown in 5% FBS, the other conditions were kept the same as those of HK-2. The cells were pretreated with C3G (50 μM) for 12 h and then the medium was replaced with medium without sugar or serum. Next, hypoxia-reoxygenation (H/R) injury was induced by exposing the cells to hypoxic conditions (1% O_2_, 5% CO_2_, and 94% N_2_) for 6 h, followed by reoxygenation under normoxic conditions (5% CO_2_ and 95% air, reoxygenation) for 24 h in DMEM with 10% FBS (H/R group). The cells were transfected with small interfering RNA (siRNA) against AMPK for 6 h by using Lipofectamine 2000 reagents (Thermo Fisher, USA), washed with phosphate buffer for 3 times and cultured normally for 24–48 h before being handled.

### Cell viability assay

Both HK-2 cells and 52E cells were plated in a 96-well plate at a density of 5000 cells per well. To evaluate the effect of C3G on ferroptosis, cell viability was tested by a Cell Counting Kit-8 (AR1199, Boster, Wuhan, China). To eliminate the side effect of agents, each well was washed by phosphate-buffered saline for twice and then CCK8 reagent was diluted with DMEM to an appropriate concentration. After incubated at 37 °C for one and a half hours, optical density (OD) values were measured at 450 nm.

### Measurement of Fe2 + , GSH, SOD, MDA and LDH

The kidney tissues or cells were harvested following the manufacturer’s instructions. The supernatants gained after centrifugation were then applied to detection of ferrous iron (Fe^2+^), glutathione (GSH), malondialdehyde (MDA), provided by Ferrous iron Colorimetric Assay Kit (E-BC-K773-M, Elabscience, Wuhan, China), the total Glutathione Assay Kit (S0052) and Lipid peroxidation malondialdehyde (MDA) assay kit (S0131M) purchased from Beyotime Biotechnology (Shanghai, China). Thus it is the same with superoxide dismutase (SOD) and lactate dehydrogenase (LDH), assessed by SOD Assay Kit (A001-3–2, Jiancheng, China) and LDH Assay Kit (A020-2–2, Jiancheng, China). Finally, the optical density was evaluated via an automated microplate spectrophotometer.

### Western blot analysis

In brief, the kidney tissues and cells were homogenized by RIPA, where the supernatant of the protein was obtained. Then the concentration of each sample was determined by using BCA Protein Assay Kit provided by Thermo Fisher. Next, an equal amount of protein samples was boiled for 10 min, electrophoresed in 10% SDS polyacrylamide gel, and transferred onto PVDF membranes (Millipore, Billerica, MA, USA). Apart from these, the membrane was blocked by 5% non-fat milk at room temperature for 1 h and subsequently incubated with the primary antibodies at 4 °C overnight. After being washed and incubated for 1 h at room temperature with the HRP-conjugated secondary antibodies with ECL chemiluminescent system.

### Real time PCR analysis

Total RNA from cells were extracted using Trizol (Invitrogen) and RNA (500 ng) was used to be reversed and transcribed for cDNA synthesis. Then quantitative PCR (qPCR) was used to assess the relative expression level of genes compared with actin. The primer sequence used in this study were quoted from what previously reported (Li et al. 2020; Qin et al. 2018b; Wang et al. 2021a).

### Statistical analysis

The significance of experimental results was evaluated by Student's t-test to compare two groups or by one-way ANOVA with Dunnett's multiple comparison tests. Immunofluorescence and grayscale analysis of western blot bands were performed semiquantitative analysis using Image J. The results were expressed as mean ± SD. *P* < 0.05 means statistically significant, with 3–6 samples in each group.

## Results

### C3G inhibited RTEC ferroptosis

**Fig. 1:**
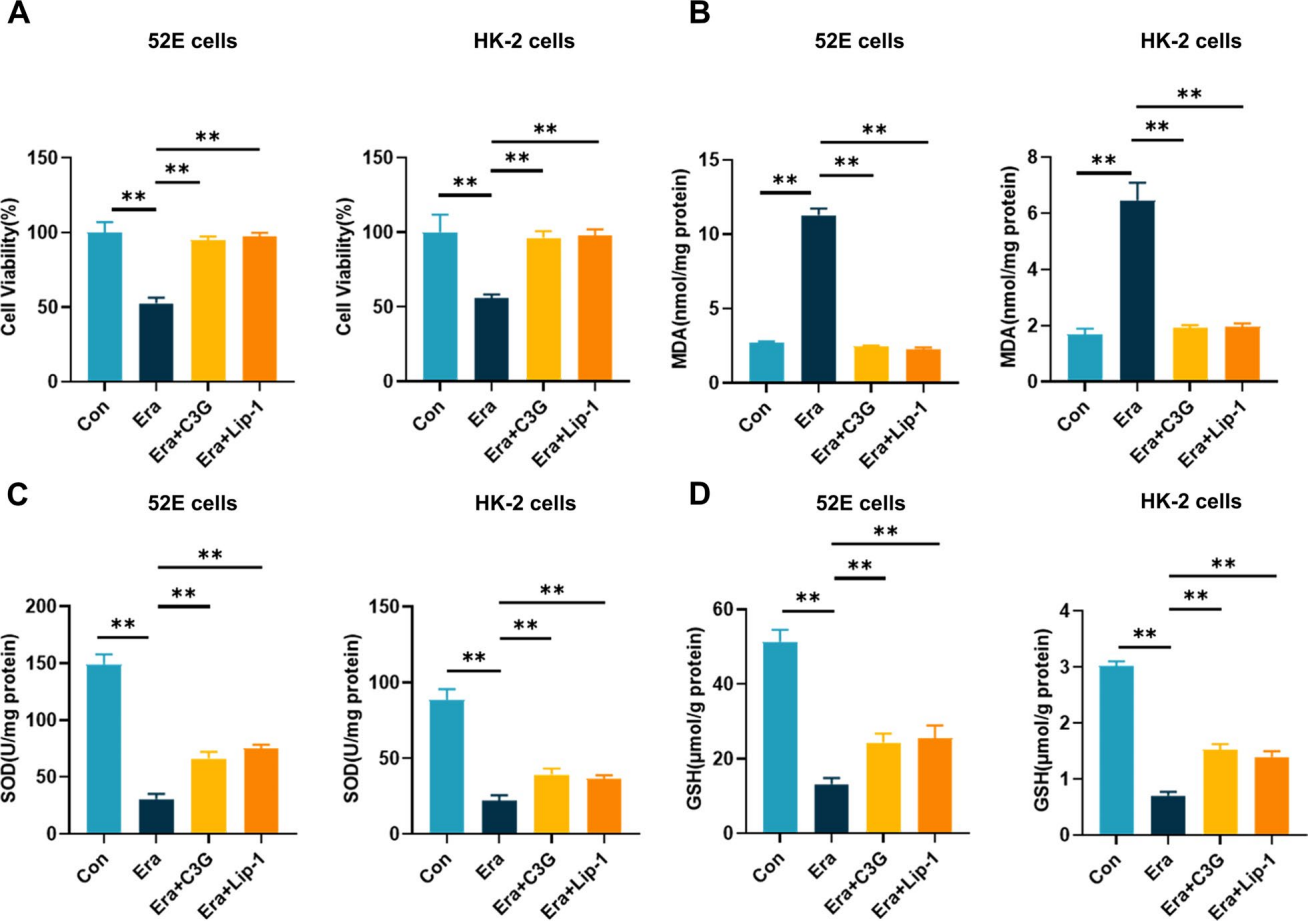
C3G markedly ameliorated ferroptosis in 52E cells and HK-2 cells. A Cell viability determined by CCK-8 cell viability assay. (n = 6). The cells were treated with drugs for 24 h; **B**–**D** The levels of MDA, SOD and GSH of different groups in 52E cells and HK-2 cells respectively. (n = 6). The cells were treated for 24 h. *p < 0.05, **p < 0.001. *C3G* cyanidin-3-glucoside, *CCK-8* cell counting kit-8; *MDA* malondialdehyde, *SOD* superoxide dismutase; *GSH* glutathione, *HK-2* human proximal tubule epithelial cells

We used Era, a ferroptosis inducer that inhibits the cystine/glutamate antiporter, to induce ferroptosis in NRK-52E cells (52E cells) and HK-2 cells. Unexpectedly, we found that C3G could protect cells from death, similar to Lip-1, an inhibitor of ferroptosis (Fig. 1A). To further verify the protective effect of G3G on ferroptosis, we examined the lipid peroxidation-associated products that are as a hallmark of ferroptosis in cells. As shown in Fig. 1B, treatment with C3G or Lip-1 abolished the adverse effect of Era on MDA levels. In contrast, the levels of GSH and SOD, which protect against oxidation and maintain homeostasis, were reduced during ferroptosis, and were rescued by C3G and Lip-1 (Fig. 1C, D). There was no difference between MDA, SOD or GSH levels after treatment with C3G or Lip-1. These experiments showed that C3G might be a ferroptosis inhibitor and additional investigations were needed to confirm this conclusion.

### C3G protected cells from H/R-induced injury in vitro

**Fig. 2:**
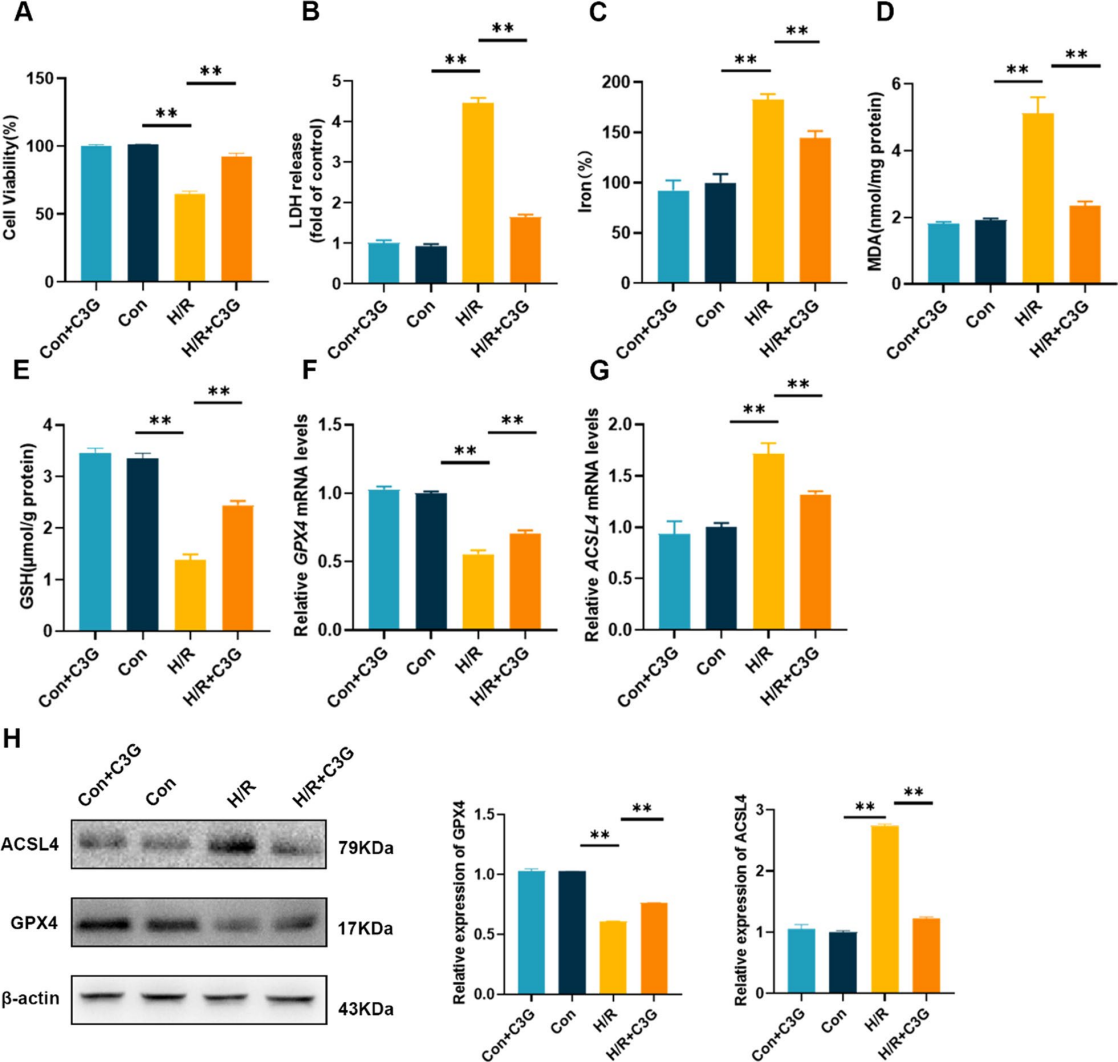
The effect of C3G on H/R-mediated ferroptosis in HK-2 cells. **A** Cell viability determined by CCK-8 cell viability assay. (n = 6). The cells were exposed to hypoxia and reoxygenation (H/R); **B** LDH release was assessed by LDH assay kit in different groups. (n = 6); **C**–**E** The levels of iron, MDA and GSH of different groups in HK-2 cells. (n = 6); **F**, **G** RT-PCR results of GPX4 and ACSL4 in HK-2 cells. (n = 6); **H** Western blot results of GPX4 and ACSL4 in HK-2 cells. (n = 3). *p < 0.05, **p < 0.001. *C3G* cyanidin-3-glucoside, *H/R* hypoxia/reoxygenation, *HK-2* human proximal tubule epithelial cells, *CCK-8* cell counting kit-8, *LDH* lactate dehydrogenase, *MDA* malondialdehyde, *GSH* glutathione, *RT-PCR* reverse transcription polymerase chain reaction, *GPX4* glutathione peroxidase 4, *ACSL4* acyl-CoA synthetase long chain family member 4

A large body of evidence has indicated that ferroptosis occurs in AKI, and has a dominant role in RTECs after I/R injury, compared with other types of programmed cell death, such as necroptosis and pyroptosis (Zhao et al. 2020; Linkermann et al. 2014; Ding et al. 2020). Based on the inhibitory effect of C3G on Era-induced ferroptosis, we investigated the specific effects of C3G on AKI. We established in vitro H/R model and an in vivo I/R model as others previously described (Ren et al. 2020; Ge et al. 2017; Liu et al. 2014, 2013). Our results showed that C3G had no cytotoxic effect on cells. C3G exerted a protective effect when cells were exposed to H/R, as indicated by improved cell survival rates and reduced the release of LDH (Fig. 2A, B). Next we observed changes in the iron levels, since the accumulation of iron is indispensable for ferroptosis. C3G reversed the increase iron levels in the H/R group (Fig. 2C). Moreover, C3G could decrease the levels of MDA induced by H/R and increase the expression of GSH, similar to its effect in the presence of Era (Fig. 2D, E). Next, we assessed the relative mRNA (Fig. 2F, G) and protein (Fig. 2H) expression levels of GPX4 and ACSL4, which are two vital enzymes responsible for ferroptosis. The results indicated that the impaired expression of GPX4 was rescued by C3G, while C3G treatment inhibited the upregulated expression of ACSL4.

### C3G attenuated I/R-induced AKI in vivo

**Fig. 3:**
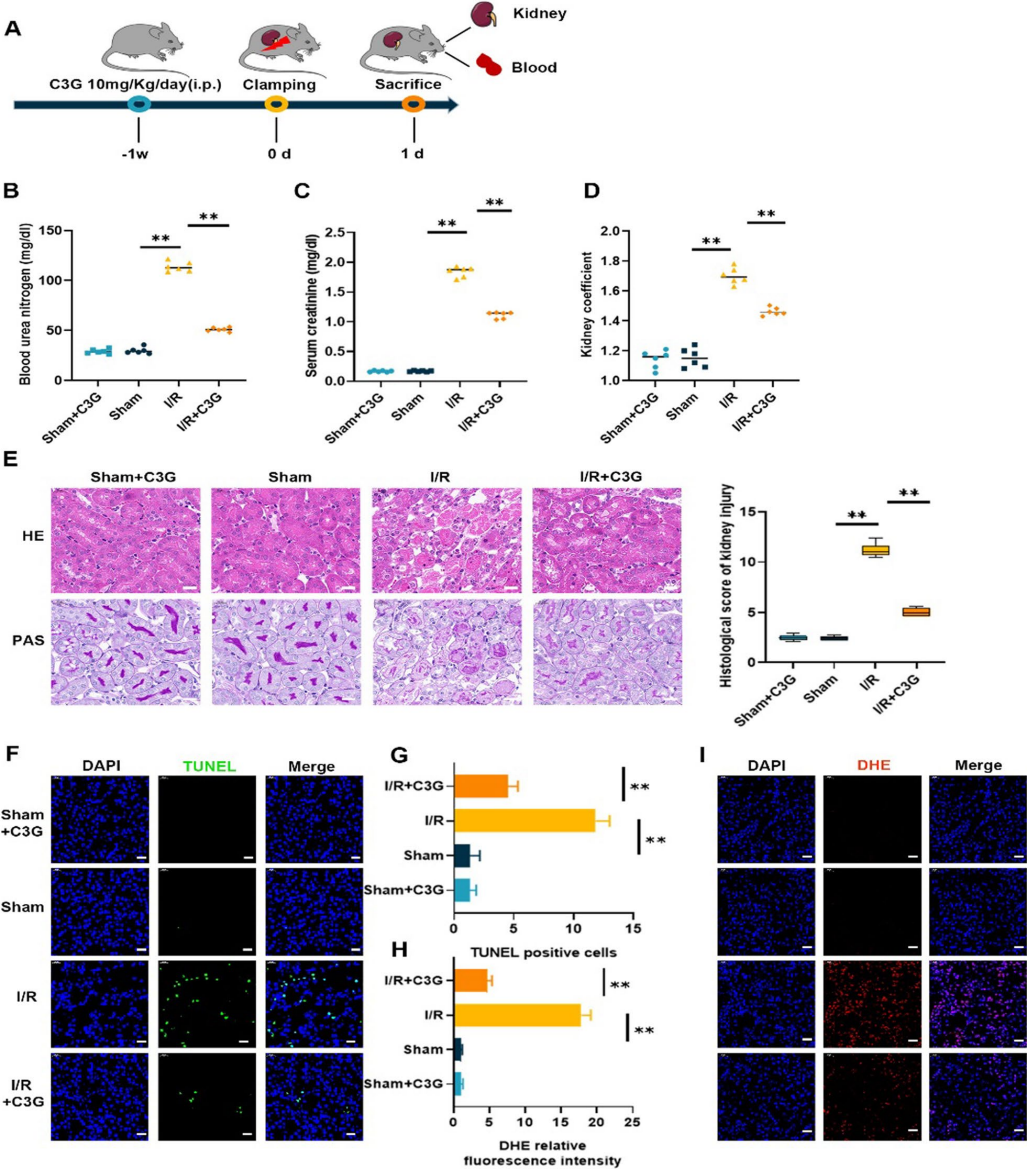
Treatment of C3G attenuated I/R-AKI in vivo. **A** Schematic of renal I/R generation and treatment of C3G for one week in advanced; **B**–**D** BUN, Scr levels and kidney coefficient. Kidney coefficient = Kidney weight/body weight × 100. (n = 6); **E** Representative micrographs of HE and PAS staining of kidney from sham + C3G, sham, I/R and I/R + C3G group. The pathological scores of kidney injury were graded. Scale bars = 20 μm. (n = 6); **F**, **G** Representative terminal deoxynucleotidyl transferase dUTP nick-end label- (TUNEL-) stained sections of different groups in kidney. Scale bars = 20 μm. (n = 6); **H**, **I** Representative immunofluorescence images of ROS were captured and analyzed by the ratio of ROS positive cells/total cells. Scale bars = 20 μm. (n = 6). *p < 0.05, **p < 0.001. *C3G* cyanidin-3-glucoside, *I/R-AKI* ischemia/reperfusion-induced acute kidney injury, *BUN* blood urea nitrogen, *Scr* serum creatinine, *HE* hematoxylin and eosin, *PAS* Periodic acid-Schiff, *ROS* reactive oxygen species

Based on the positive in vitro observations, we further investigated the protective effect of C3G in vivo. C3G (10 mg/kg) was intraperitoneally injected for one week before the bilateral arteries were clamped for 33 min and then the mice were sacrificed 24 h after I/R (Fig. 3A). First, we ensured that C3G alone did not induce any harmful effects on normal mice. Next, we investigated the effect of C3G on I/R-induced AKI. Consistent with the in vitro results, C3G treatment dramatically decreased the levels of BUN (Fig. 3B), and Scr (Fig. 3C) and the kidney coefficient (Fig. 3D). The pathological changes were evaluated by HE staining and PAS staining (Fig. 3E). Compared with that in the sham group, degeneration of tubular epithelium, the loss of brush borders and dilatation were observed in the I/R group, whereas, C3G effectively attenuated these injuries. TUNEL immunofluorescence staining of kidney tissue showed C3G ameliorated kidney cell apoptosis (Fig. 3F, G). In addition, because the antioxidant activity of C3G is well known, we also measured ROS levels (Fig. 3H, I), which are indicators of oxidative stress, and the results showed was drawn that C3G inhibited the increase in ROS in I/R damaged tissues.

### C3G decreased iron accumulation and reversed the expression of ferroptosis-related genes in kidney after I/R injury in vivo

**Fig. 4:**
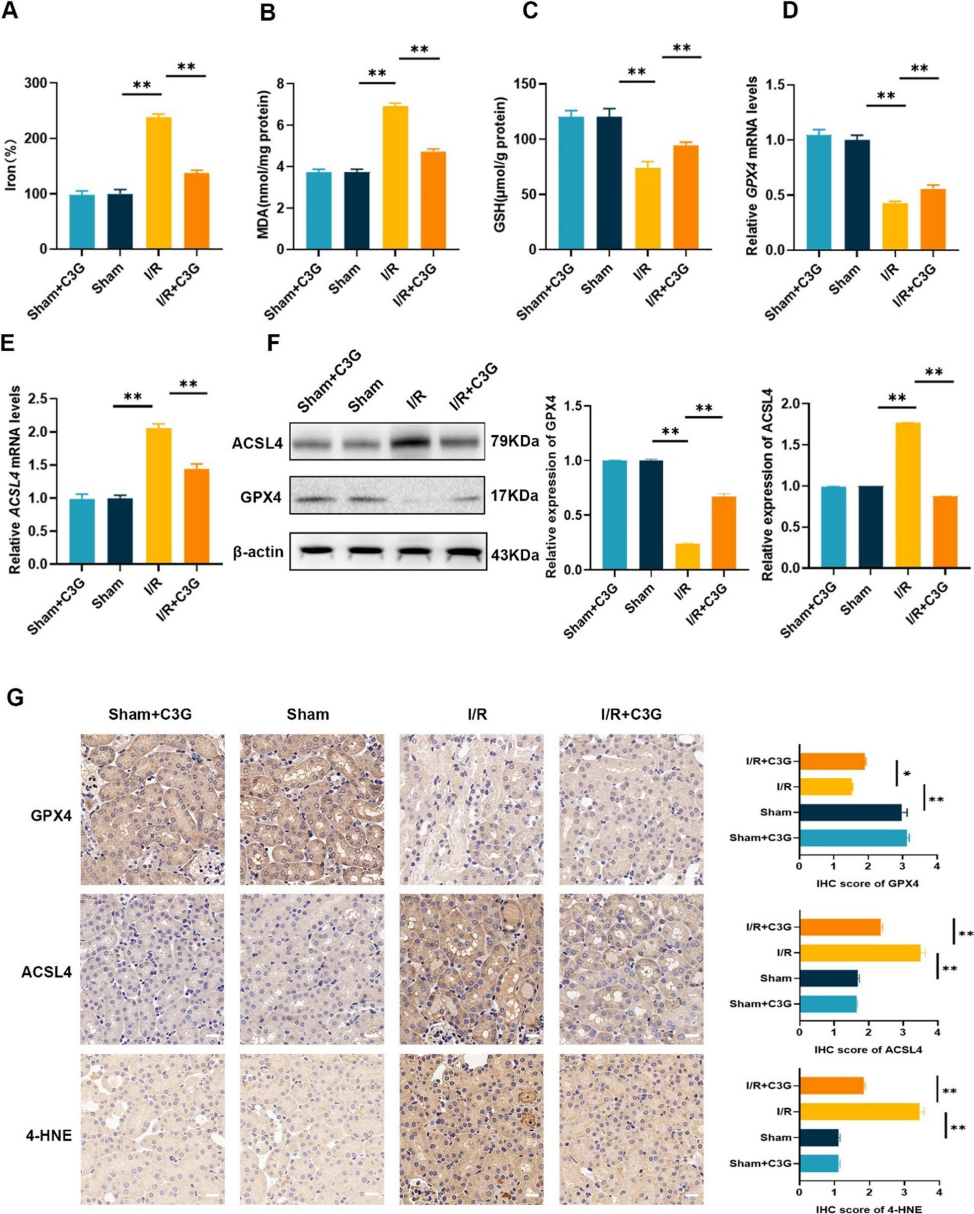
C3G mitigated ferroptosis in the kidneys from I/R-AKI mice. **A**–**C** The content of iron, MDA and GSH were determined in kidney tissues of different groups. (n = 6); **D**–**E** RT-PCR results of GPX4 and ACSL4 of sham + C3G, sham, I/R and I/R + C3G group. (n = 6); **F** Western blot results of GPX4 and ACSL4 in kidney tissues. (n = 3); **G** Representative immunohistochemistry images of GPX4, ACSL4 and 4-HNE were captured respectively and analyzed. Scale bars = 20 μm. (n = 3). *p < 0.05, **p < 0.001. *C3G* cyanidin-3-glucoside, *I/R-AKI* ischemia/reperfusion-induced acute kidney injury, *MDA* malondialdehyde, *RT-PCR* reverse transcription polymerase chain reaction, *GPX4* glutathione peroxidase 4, *ACSL4* acyl-CoA synthetase long chain family member 4, *4-HNE* 4-hydroxynonenal

Ferroptosis, which is characterized by intracellular free iron accumulation and disruption of antioxidant system, with subsequent lipid peroxidation, results in the loss of membrane integrity. Thus, we also detected iron levels. After I/R, free iron and MDA levels were significantly elevated, and this effect could be abolished by C3G (Fig. 4A, B). In contrast, the decrease in GSH was mitigated by C3G (Fig. 4C). Next, the same experiments were carried out in vivo. We measured mRNA and protein expression of GPX4 and ACSL4 with or without C3G. During the I/R, the decrease in the mRNA expression of GPX4 and increase in the mRNA expression of ACSL4 were mitigated by C3G. (Fig. 4D, E). Moreover, parallel changes were observed in the protein expression levels, as shown by western blot analysis (Fig. 4F) and immunohistochemical staining (Fig. 4G). Notably, C3G can inhibit the adverse effect of ferroptosis on AKI, in vitro or in vivo, to alleviate the cell and kidney injury, indicating that C3G is a potential drug that affects ferroptosis in the renal tubular epithelium.

### C3G inhibited ferroptosis by increasing AMPK phosphorylation

**Fig. 5:**
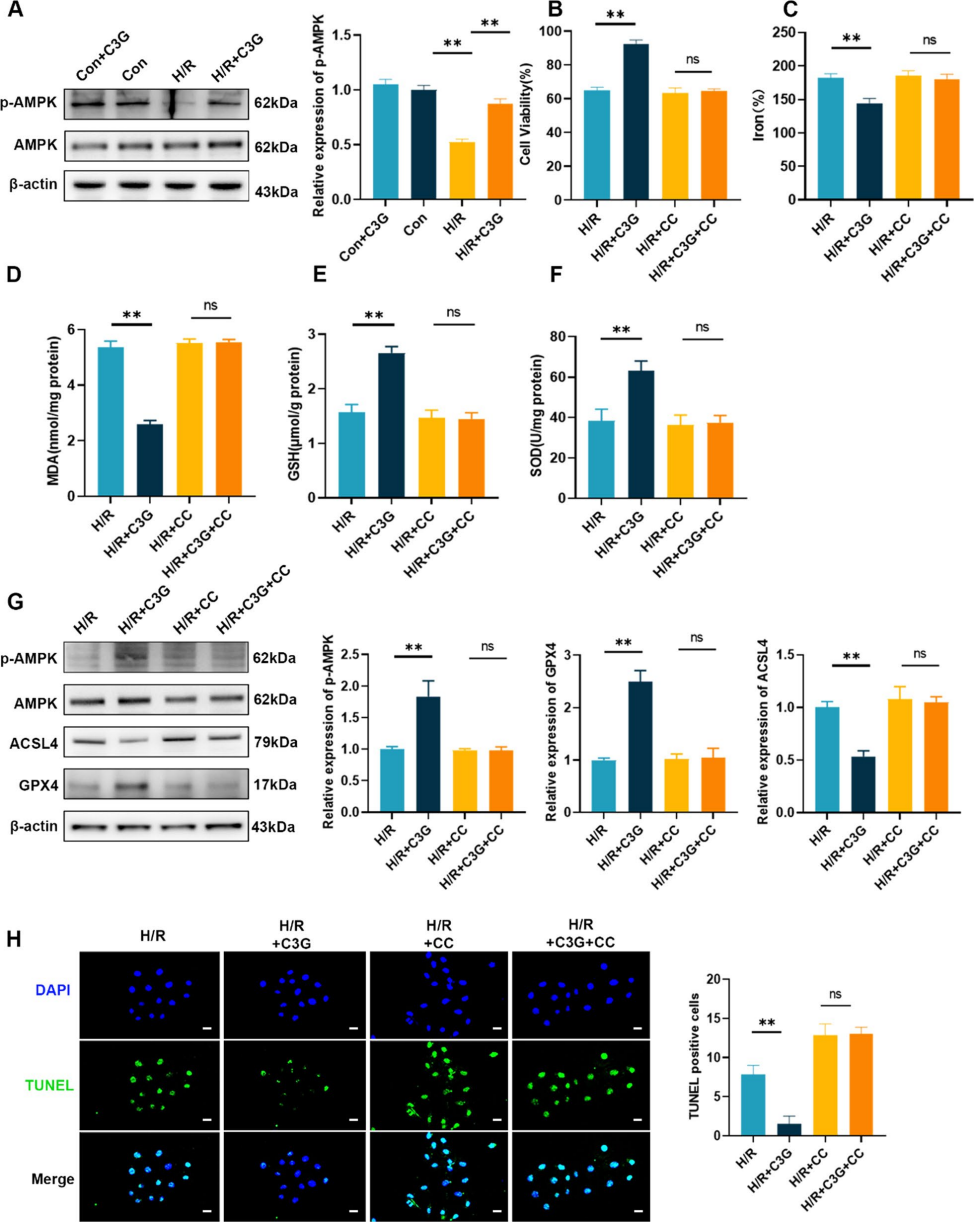
The inhibitory effect of C3G on HK-2 cell ferroptosis was dependent on AMPK activation. **A** The efficiency of activation of AMPK via C3G was analyzed by Western blot. (n = 3); **B** Cell viability determined by CCK-8 cell viability assay. (n = 6). The cells were exposed to hypoxia and reoxygenation (H/R) incubated with C3G, CC or both; **C**–**F** The content of iron, MDA, GSH and SOD were determined in HK-2 cells of different groups. (n = 6); **G** Western blot results of AMPK, GPX4 and ACSL4 in HK-2 cells with or without CC. (n = 3); **H** Representative terminal deoxynucleotidyl transferase dUTP nick-end labeling- (TUNEL-) stained sections of different groups in kidney. Scale bars = 20 μm. (n = 6). *p < 0.05, **p < 0.001. *C3G* cyanidin-3-glucoside, *HK-2* human proximal tubule epithelial cells, *AMPK* AMP-activated protein kinase, *CCK-8* cell counting kit-8, *CC* Compound C, *MDA* malondialdehyde, *GSH* glutathione, *SOD* superoxide dismutase, *GPX4* glutathione peroxidase 4, *ACSL4* acyl-CoA synthetase long chain family member 4

**Fig. 6:**
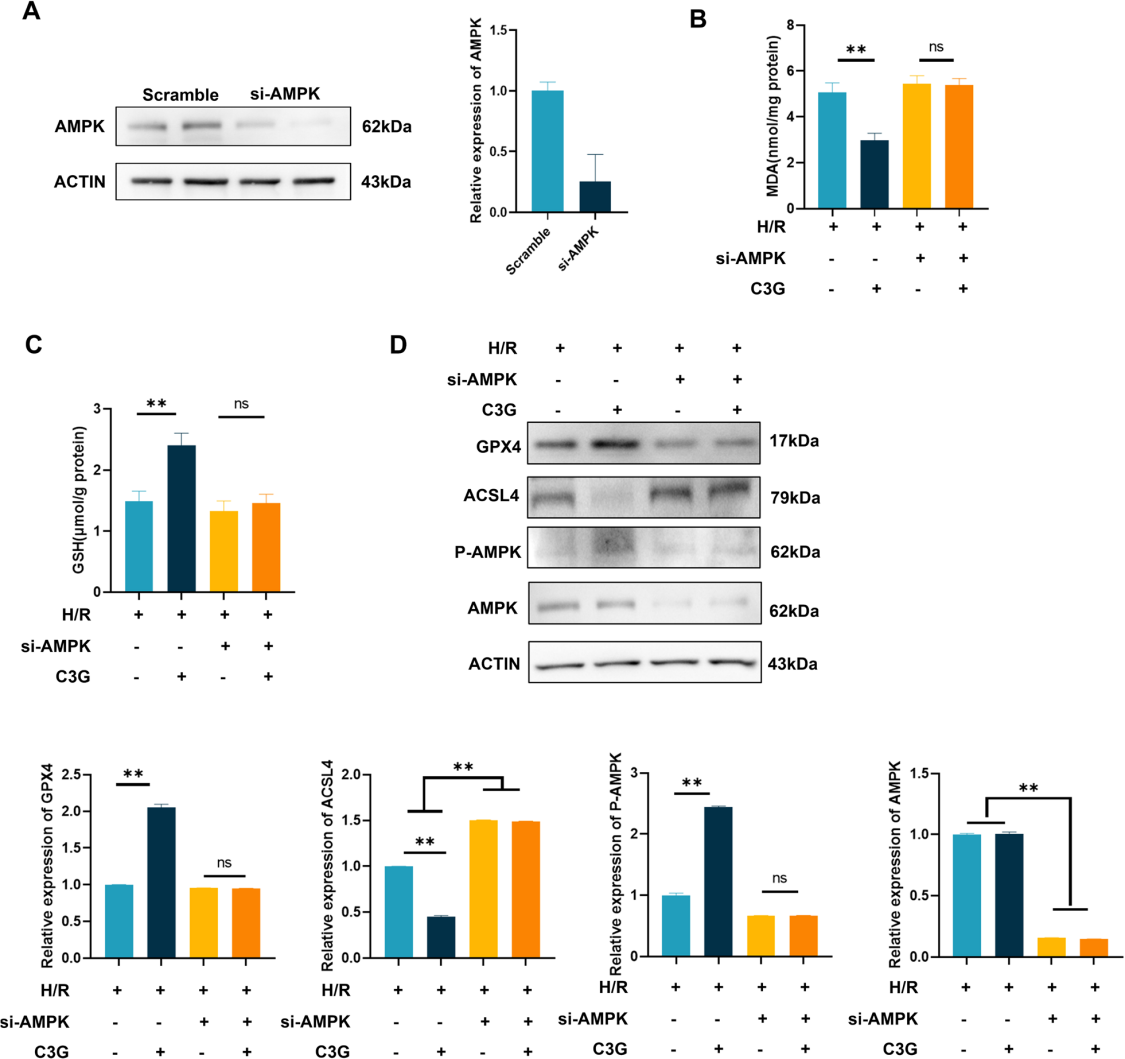
The knockdown of AMPK abrogated the protective effect of C3G against ferroptosis. **A** The efficiency of knockdown of AMPK via si-AMPK was analyzed by Western blot. (n = 3); **B**, **C** The content of MDA and GSH were determined in HK-2 cells with or without C3G after knockdown of si-AMPK under H/R condition. (n = 6); **D** Western blot results of AMPK, P-AMPK, GPX4 and ACSL4 in HK-2 cells with or without C3G after knockdown of si-AMPK under H/R condition (n = 3). *p < 0.05, **p < 0.001. *C3G* cyanidin-3-glucoside, *HK-2* human proximal tubule epithelial cells, *AMPK* AMP-activated protein kinase, *MDA* malondialdehyde, *GSH* glutathione; *GPX4* glutathione peroxidase 4, *ACSL4* acyl-CoA synthetase long chain family member 4, *H/R* hypoxia/reoxygenation

Studies have indicated an underlying relationship between C3G, AMPK and ferroptosis (Lee et al. 2020; Shan et al. 2021; Kurimoto et al. 2013; Guo et al. 2012), and we hypothesized that C3G may activate AMPK to inhibit ferroptosis in RTECs. To verify our hypothesis, in vitro experiments with HK-2 cells were performed. Under normoxia, we did not observe a shift in AMPK phosphorylation after C3G treatment. However, the decrease in AMPK phosphorylation during H/R could be reversed by C3G, suggesting the potential regulatory effect of C3G on AMPK (Fig. 5A). To confirm the association between C3G-mediated inhibition of ferroptosis and the activation of AMPK phosphorylation, we treated HK-2 cells exposed to H/R with or without CC, an inhibitor of AMPK. First, we observed that cell viability was rescued by C3G during H/R and was diminished by CC (Fig. 5B). Next, we assessed the indicators associated with ferroptosis, and the application of CC abolished the ameliorating effect of C3G on iron (Fig. 5C) and MDA (Fig. 5D) levels. In addition, we detected the effect of CC on C3G in the antioxidation regimen. Consistently, the effect of C3G on alleviating changes in GSH (Fig. 5E) and SOD (Fig. 5F) was abrogated by CC. Western blot analysis showed concomitant changes in the protein expression mentioned above, revealing that the activation of AMPK, the increased expression of GPX4 and the decrease in ACSL4 were abolished by CC (Fig. 5G). In addition, TUNEL staining revealed that the inhibitory effect of C3G on ferroptosis depends on the activation of AMPK in HK-2 cells (Fig. 5H). To further confirm our conclusion, we used small interfering RNA (siRNA) to knockdown the expression of AMPK, and we used the published sequence of si-AMPK (Yan et al. 2022). First, we determined the effectiveness of the sequence via transfection and western blot (Fig. 6A). Next, we exposed HK-2 cells to hypoxia/reoxygenation conditions and further observed the effect on MDA and GSH levels after knocking down AMPK with or without the addition of C3G. As shown in Fig. 6B, C, C3G ameliorated ferroptosis-induced injuries in the scramble group; however, in the si-AMPK group, inactivation of AMPK did not rescue the increase in MDA levels and decrease in GSH levels even in the presence of C3G, indicating that the protective effect of C3G was abrogated by knocking down AMPK under H/R conditions. Finally, we observed a change in the expression of proteins associated with ferroptosis. The WB results demonstrated that C3G increased the phosphorylation level of AMPK and expression of GPX4 and reduced ACSL4 expression in the scramble group. However, knockdown of AMPK did not upregulate the expression of P-AMPK and GPX4; moreover, it did not reduce the increase in ACSL4, even after C3G treatment (Fig. 6D). In conclusion, we drew the same conclusion as previous studies using CC. The downregulation of AMPK resulted in the inactivation of P-AMPK and eliminated C3G-mediated inhibition of ferroptosis in HK-2.

### AMPK inactivation eliminated the inhibitory effect of C3G on ferroptosis in vivo

**Fig. 7:**
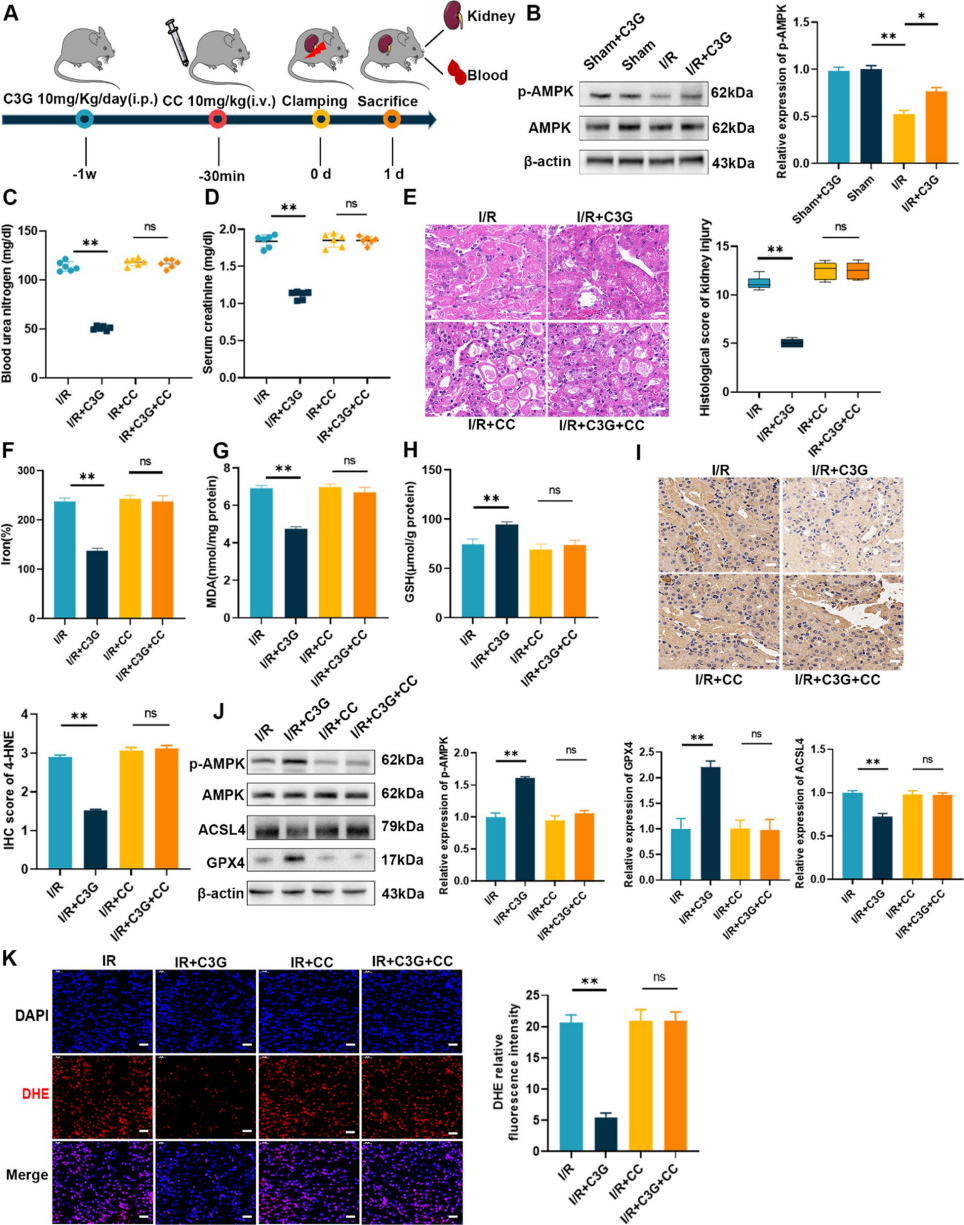
C3G alleviated I/R-AKI via activation of AMPK in vivo. **A** Schematic of renal I/R generation and treatment of C3G and CC at different period; **B** The protein level of activation of AMPK via C3G was analyzed by Western blot. (n = 3); **C**, **D** BUN and Scr levels in I/R-AKI mice. (n = 6); **E** Representative micrographs of HE staining of kidney from I/R, I/R + C3G, I/R + CC, I/R + C3G + CC group. The pathological scores of kidney injury were graded. Scale bars = 20 μm. (n = 6); **F**–**H** The content of iron, MDA, GSH were determined in kidney tissues of different groups. (n = 6). **I** Representative immunohistochemistry images of 4-HNE were captured respectively and analyzed. Scale bars = 20 μm. (n = 3); **J** Western blot results of AMPK, GPX4 and ACSL4 in kidney with or without CC. (n = 3); **K** Representative immunofluorescence images of ROS were captured and analyzed by the ratio of ROS positive cells/total cells. Scale bars = 20 μm.*p < 0.05, **p < 0.001. *C3G* cyanidin-3-glucoside, *I/R-AKI* ischemia/reperfusion-induced acute kidney injury, *AMPK* AMP-activated protein kinase, *CC* Compound C, *BUN* blood urea nitrogen, *Scr* serum creatinine, *HE* hematoxylin and eosin; *MDA* malondialdehyde; *GSH* glutathione, *4-HNE* 4-hydroxynonenal, *GPX4* glutathione peroxidase 4, *ACSL4* acyl-CoA synthetase long chain family member 4, *4-HNE* 4-hydroxynonenal, *ROS* reactive oxygen species

**Fig. 8:**
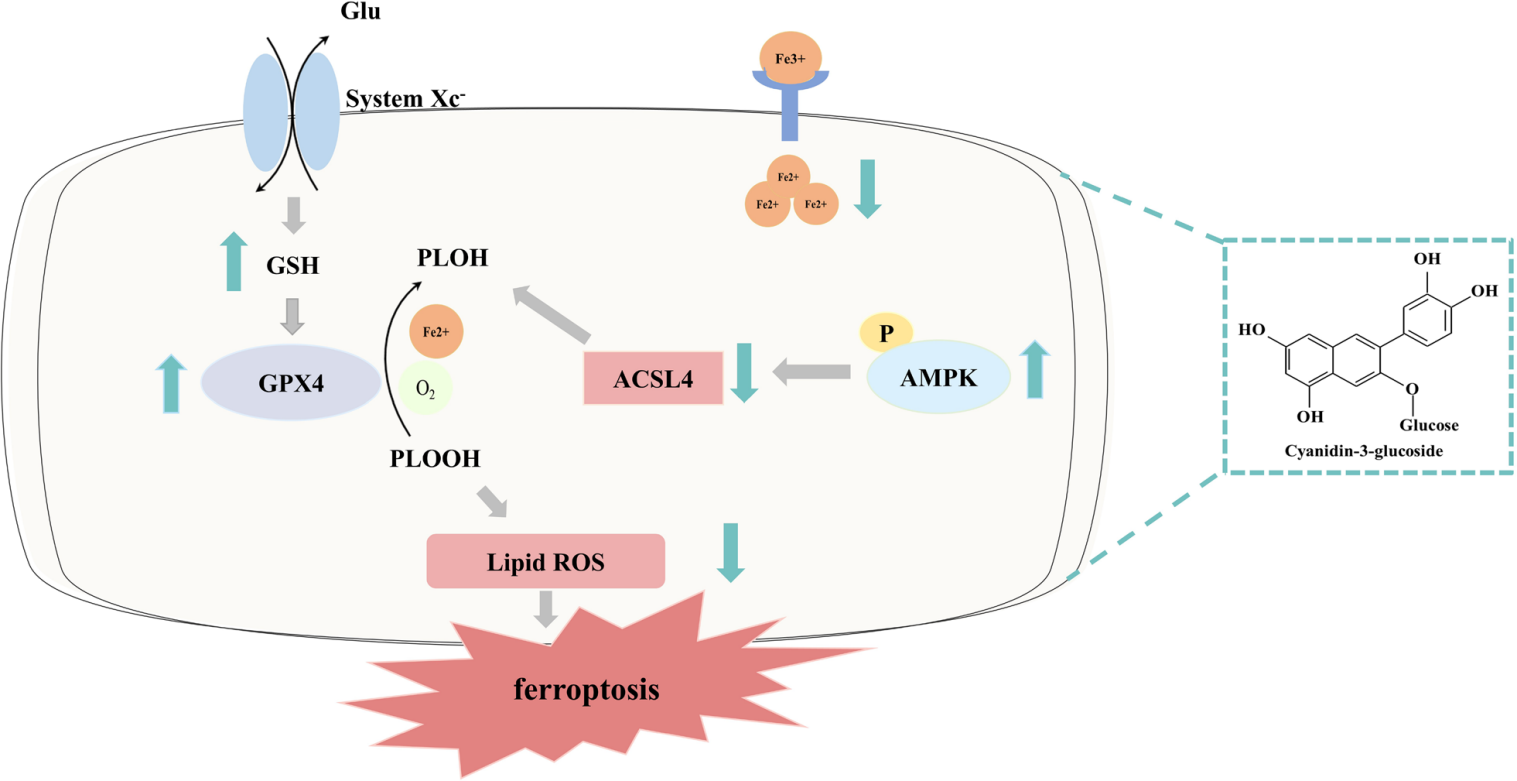
A proposed scheme demonstrating the protective effect of C3G against AKI. C3G decreased free iron accumulation and lipid peroxidation products, increased the levels of GSH and the expression of GPX4 and downregulated the expression of ACSL4 to inhibit ferroptosis in the renal tubular epithelial cells during AKI, which is dependent on the activation of AMPK

We performed experiments to determine whether C3G affected the activation of AMPK in vivo. We intravenously injected CC 30 min before the operation to inhibit the activation of AMPK. The mice were sacrificed after one day of reperfusion (Fig. 7A). Consistent with the in vitro results, treatment with C3G did not affect the activation of AMPK in the sham-operated group but successfully prevented the decrease in AMPK phosphorylation in the I/R group (Fig. 7B). Similar to our observations in HK-2 cells, we found that the C3G-mediated protective effect on blood urea nitrogen, serum creatinine and renal damage was abolished by CC (Fig. 7C–E). To elucidate the underlying relationship between AMPK activation and ferroptosis in vivo, we measured ferroptosis-related indicators. CC eliminated the alleviation of changes in iron and MDA levels (Fig. 7F, G) and abolished the restorative effect on GSH (Fig. 7H). Injection of CC inhibited the activation of AMPK, which counteracted the effect of C3G, as shown by the protein expression levels of ACSL4 and GPX4 (Fig. 7I). Next, we assessed the alterations in 4-HNE via immunohistochemical staining, and the application of CC suppressed the protective effect of C3G, which reduced 4-HNE staining (Fig. 7J). In addition, DHE staining suggested that ROS restoration by C3G could be reversed by the inactivation of AMPK. After treatment with CC, the attenuation caused by C3G disappeared (Fig. 7K). Overall, C3G exerted a nephroprotective effect against ferroptosis through the activation of AMPK in vitro and in vivo (Fig. 8).

## Discussion

AKI still lacks effective prevention and treatment strategies. Ferroptosis is the dominant form of programmed cell death, and there is a pressing need for novel therapeutic approaches. In this study, we reported for the first time that C3G attenuated ferroptosis by activating AMPK to protect against I/R-AKI and highlighted the following findings: 1) C3G markedly ameliorated Era-induced ferroptosis, which was characterized as a reduction in lipid peroxide and MDA production and an increase in GSH levels, as well as an improvement in mitochondrial morphology, resulting in nearly equivalent effects as Lip-1, a selective ferroptosis inhibitor (Li et al. 2019); 2) the inhibitory effect of C3G on ferroptosis was observed in I/R-AKI models in vitro and in vivo, which was characterized by the reversion of excessive intracellular free iron accumulation and ROS production, an increase in GPX4 expression and GSH levels, and a decrease in ACSL4 expression and lipid peroxidation; and 3) C3G activated the AMPK pathway in RTECs in I/R-AKI models in vitro and in vivo, but the selective AMPK inhibitor CC and knockdown of AMPK abolished the inhibitory effect of C3G on ferroptosis.

The renal protective effect of C3G on diabetic nephropathy has gained wide attention (Qin et al. 2018a; Zheng et al. 2020), but only one report recently focused on renal I/R injury. Li et al. (2021) found that C3G played a renal protective role in I/R-AKI by inhibiting inflammation, oxidative stress, apoptosis and lipid peroxidation, but they did not mention the mechanism of ferroptosis inhibition. In fact, flavonoids, which are a class of polyphenolic compounds, can inhibit lipid peroxidation caused by iron overload because of their iron chelating and antioxidant effects (Wang et al. 2021b). We previously confirmed that the polyphenol compound polydatin inhibits inflammation, oxidative stress and ferroptosis in I/R-AKI and cisplatin-AKI models (Liu et al. 2015; Meng et al. 2016; Zhou et al. 2022). C3G, which is a flavonoid, should theoretically inhibit ferroptosis. Recently, Setapramoten et al. (Settapramote et al. 2021) discovered the antioxidant and protective effect of C3G on an iron dextran-loaded mouse model. In the same year, Shan X et al. confirmed the antiferroptotic effect of C3G in myocardial I/R injury (Zhang et al. 2021b). Our results showed that C3G could significantly rescue intracellular free iron accumulation, GSH depletion and ROS production induced by H/R and I/R in I/R-AKI models in vitro and in vivo, suggesting the antiferroptotic effect of C3G on I/R-AKI.

Erastin, which is a potent selective system Xc¯ inhibitor, depletes intracellular GSH by preventing cystine uptake, which in turn leads to a reduction in the activity of several GSH-dependent antioxidant enzymes, including GPX4, and the activation of lipoxygenases (LOXs). GPX4 is a lipid enzyme that catalyzes the conversion of GSH to glutathione disulfide (GSSG) in the oxidation reaction, removes excess peroxides and subsequently alleviates the peroxidation of polyunsaturated fatty acids in the membrane(Zhang et al. 2021a). In this study, we determined that C3G significantly alleviated Era-induced cell death in NRK-52E cells and HK-2 cells. Furthermore, C3G significantly reversed the decrease in GPX4 expression induced by H/R or I/R. These results indicate that C3G may play an antiferroptotic role at least partially by regulating the system Xc¯-GSH-GPX4 axis.

AMPK is a serine/threonine kinase whose activation leads to the restoration of energy levels. With respect to lipids, AMPK is directly phosphorylated to inhibit acetyl coenzyme A (CoA) carboxylase (ACC), which is the first enzyme in fatty acid synthesis that converts acetyl-CoA to malonyl-CoA. Energy stress-mediated AMPK activation suppresses ferroptosis by inhibiting ACC (Lee et al. 2020; Currais et al. 2022). ACSL4 can activate and incorporate free polyunsaturated acyl tails (PUFAs) into phospholipids to generate phospholipids with PUFAs (PL-PUFAs), which are substrates for peroxidation during ferroptosis, and so it is recognized as a positive regulator of ferroptosis(Hadian and Stockwell 2020). During the ischemic period, intracellular adenosine triphosphate (ATP) levels fall significantly (Pefanis et al. 2019), and the expression of activated AMPK increases to compensate. During the reperfusion period, the decrease in AMPK phosphorylation cannot maintain homeostasis, and subsequently, increases in renal damage and blood urea nitrogen are observed (Lee et al. 2020). Ten years ago, several reports focused on C3G-mediated regulation of fatty acid metabolism by activating the AMPK pathway, although these researchers did not examine whether ferroptosis was involved (Wei et al. 2011; Guo et al. 2012). The results of this study confirmed for the first time that C3G significantly reduced ACSL4 expression in RTECs induced by H/R or I/R, which was accompanied by decreased lipid peroxidation, and this effect was reversed by the AMPK inhibitor CC, suggesting that C3G at least partially reduced the production of peroxide substrates by activating the AMPK pathway and inhibiting ACSL4 expression.

Iron chelating agents, including desferrioxamine, deferiprone and deferasirox, have been proven to have antiferroptotic effects, but they are only used clinically to treat the iron overload in thalassemia caused by excessive blood transfusions (Kontoghiorghes and Kontoghiorghe 2020). In addition, these agents also have the disadvantages of deleterious side effects, poor patient compliance and high prices. Ferroptosis-specific inhibitors, such as ferrostatin-1 (Fer-1) and liproxstatin-1 (Lip-1), exert protective effects against cell and animal injury, which is sufficient for scientific research, but they are still a long way from clinical use (Zhao et al. 2020; Martin-Sanchez et al. 2017; Wang et al. 2021a; Li et al. 2019). C3G is widely distributed in a multitude of vegetables and fruits, and compared with other agents, C3G is more available and economical (Zhang et al. 2020; Deng et al. 2013). Apart from this, its safety has been determined in humans, and the consumption of anthocyanin-rich acai juice and pulp dramatically increase plasma antioxidant capacity (Mertens-Talcott et al. 2008). Therefore, our research results on the antiferroptotic effect of C3G open a new avenue for future treatment of AKI with C3G.

## Conclusion

In summary, this study confirmed the nephroprotective effect of C3G against ferroptosis by promoting AMPK activation in an H/R cell model and an I/R-AKI animal model. We found that C3G was a much more economical, safe and stable agent that could inhibit ferroptosis by regulating the system Xc¯-GSH-GPX4 axis, activating the AMPK pathway and reducing ACSL4-dependent lipid biosynthesis, providing novel insight and a potential strategy for the treatment of AKI.

## Data Availability

All data related to this paper may also be requested from the corresponding authors (email: xjsnlhb@fmmu.edu.cn).

## Abbreviations

C3G: Cyanidin-3-glucoside
I/R-AKI: Ischemia/reperfusion-induced acute kidney injury
AMPK: AMP-activated protein kinase
CC: Compound C
BUN: Blood urea nitrogen
Scr: Serum creatinine
HE: Hematoxylin and eosin
MDA: Malondialdehyde
GSH: Glutathione
4-HNE: 4-Hydroxynonenal
GPX4: Glutathione peroxidase 4
ACSL4: Acyl-CoA synthetase long chain family member 4
ROS: Reactive oxygen species
HK-2: Human proximal tubule epithelial cells
AMPK: AMP-activated protein kinase
CCK-8: Cell counting kit-8
SOD: Superoxide dismutase
